# How to Handle Your Dragon: Does Handling Duration Affect the Behaviour of Bearded Dragons (Pogona Vitticeps)?

**DOI:** 10.3390/ani10112116

**Published:** 2020-11-15

**Authors:** Victoria R. Stockley, Anna Wilkinson, Oliver H.P. Burman

**Affiliations:** School of Life Sciences, University of Lincoln, Brayford Way, Brayford Pool, Lincoln LN6 7TS, UK; victoria@animalsenses.co.uk (V.R.S.); awilkinson@lincoln.ac.uk (A.W.)

**Keywords:** bearded dragon, reptile welfare, reptile behaviour, response to novelty, Pogona vitticeps

## Abstract

**Simple Summary:**

Keeping reptiles as pets has become relatively common; therefore, it is important for us to understand more about how different aspects of their life as pets might affect their welfare. Very little is known about the effects of handling on reptiles, particularly the type of gentle handling (i.e., without restraint) likely to be experienced by pet animals. Therefore, this study investigated whether the amount of time that bearded dragons, a commonly kept lizard species, experienced gentle handling impacted on their behaviour in tests that give insight into how they may be feeling. We found that longer durations of handling appeared to increase stress-related behaviour slightly. This finding suggests that handling bearded dragons for longer periods of time, even in a gentle way, may be mildly stressful for them. However, we do not know whether animals would become accustomed to handling for longer periods with more experience.

**Abstract:**

Reptiles are popular as pets and it is, therefore, important to understand how different aspects of housing and husbandry impact on their behaviour and welfare. One potential cause of stress in captive reptiles is interaction with humans; in particular, the effect of handling. However, little research on handling has been carried out with reptiles, particularly relating to the type of gentle handling likely to be experienced by pet animals. The aim of this study was therefore to determine whether the amount of time that bearded dragons (Pogona vitticeps), a commonly kept pet species, experienced gentle handling induced no or differing levels of anxiety, as reflected in their subsequent behavioural response to novelty. We found that there appeared to be a mildly aversive effect of handling time on subsequent behavioural response to novelty. Longer durations of handling (5 min or 15 min) appeared to increase anxiety-related behaviour, with handled animals showing more frequent tongue flicking behaviour when they experienced a novel environment and reduced time spent in close proximity to a novel object. These results suggest that handling bearded dragons, even in a gentle way, may increase their anxiety. However, it is not yet known whether animals may habituate to handling for longer periods if provided with additional experience.

## 1. Introduction

Reptiles are relatively common as pets worldwide. For example, of just under 21 million pets (excluding ornamental fish) in the UK, an estimated 700,000 are reptiles [1], and, of a total of 251 million pets in the US, an estimated 6 million are reptiles [2]. It is thought that a significant proportion of these pet reptiles may be experiencing poor welfare, often due to a lack of knowledge about their basic needs or the ability to provide suitable housing based on ecological requirements. In a pet census undertaken by the Blue Cross [3], it was found that almost half of all reptile owners had experienced unexpected problems with their pet(s). Issues typically included owners failing to recognise signs of illness, an incorrect assumption of hardiness and a lack of understanding of suitable husbandry equipment and its use [4]. In comparison to the considerable amount of research concerning the welfare of mammalian and avian species, there are relatively few studies that have focused on reptiles [5] making clear recommendations to owners challenging.

One important potential cause of stress in captive animals is direct interaction with humans; in particular, handling [6,7,8]. For instance, many pet rabbits show fear behaviours (including fear aggression) when lifted from the ground [9]. However, sympathetic handling that takes into account species-specific behaviour and focuses on a positive interaction can reduce or eliminate these negative impacts and, in some cases, can even be beneficial. For example, laboratory mice show reduced handler avoidance and anxiety related postures when picked up using a cupped hand or short tunnel compared to the more aversive technique of using the base of the tail [10]. Pigs also benefit from sympathetic contact, exhibiting faster voluntary approach to human handlers and approaching a novel object faster after positive human interactions [11], suggesting a general reduction in anxiety.

In comparison to mammalian species, little research on human-animal interactions has been carried out for reptiles (see Borgmans [12]), and generally with less emphasis on the behavioural changes that can be direct indicators of poor welfare [13]. Handling has been identified as a potentially (negative) stressor for reptiles in a handful of studies, mostly focusing on physiological responses to potentially aversive handling that incorporates restraint. For example, Kalliokoski et al. [14] investigated the effects of handling on previously unhandled green iguanas (*Iguana iguana*). After manually restraining the iguanas for five minutes each day for eight days, they found significantly higher levels of faecal corticosterone metabolites, suggesting a stress response to the handling/restraint, although there were no significant changes in behaviour. Handling/restraint has also been found to increase corticosterone levels in several other species (e.g., rattlesnakes (*Crotalus atrox*) [15] and alligators (*Alligator mississippiensis*) [16]).

The quantity (i.e., duration), rather than the quality (i.e., technique), of handling is also an important consideration; however, little research has been carried out in this specific area. A related study investigating the physiological impact of capture and restraint duration on sea turtles (*Lepidochelys kempii*) identified a significant increase in plasma corticosterone levels with the increasing time (0, 30, 60 mins) that individuals were kept turned on their backs. This revealed a stress response to acute handling that was sensitive to the duration of the experience [17]. In contrast, no such increase in corticosterone was observed for Eastern bearded dragons (*Pogona barbata*) with the increased time that they spent confined in a cloth bag after capture (0, 3.5, 24 h) [18].

A study that assessed both physiological and behavioural responses in order to compare different handling techniques (gentle handling, manual restraint, container restraint) found no effect in skinks (*Tiliqua scincoides*) but an increase in corticosterone in royal pythons (*Python regius*) when restrained in a container [19], albeit in a study with small sample sizes. Cannon et al. [20] looked at behavioural and temperature changes in Central bearded dragons (*Pogona vitticeps*) before and after being held and manipulated for 20 min, compared to a control group without handling. A behavioural difference was seen, with the bearded dragons spending significantly less time hiding after being handled, but there was no corresponding change in temperature. From these studies it is clear that further investigation into the behavioural impact of handling techniques typically used by pet owners is important to give a more fully formed picture of how handling affects reptile behaviour and welfare. Furthermore, it is essential to investigate this issue using gentle handling techniques in animals that are experienced with being handled, because this would better reflect the situation that many pet reptiles experience.

The aim of this study was to determine whether the amount of time that bearded dragons (*Pogona vitticeps*) are gently handled causes or impacts anxiety, as reflected in their subsequent behavioural response to novelty (e.g., [11]). It is predicted that, if the handling is stressful for the animals, then the longer an individual is held the more anxiety that will be induced, and, therefore the more its subsequent exploratory behaviour will be suppressed.

## 2. Materials and Methods

The research was approved by the University of Lincoln’s ethics committee (UoL2018-CAB-002).

### 2.1. Subjects

Bearded dragons are diurnal agamid lizards native to central Australia, growing to ca. 46–61 cm as mature adults. They are popular as pets [21], possibly due to their relatively small size and ease of handling compared to other lizards. Bearded dragons have also been increasingly used as subjects in reptile biology research (e.g., [22,23]).

Thirteen captive-bred adult lizards of both sexes were used in this study. They were part of a captive population held at the University of Lincoln, UK. Animals were housed individually (n = 7) or in pairs (n = 6) in vivaria (150 × 60 × 45 cm) containing a UV strip bulb along the back wall and a heat lamp at one end. They had sheltered areas and climbing branches throughout the vivarium, ad libitum access to water, were fed leafy greens once a day and received live invertebrate food (i.e., locusts and crickets) three times per week. The vivariums were in a temperature-controlled room and had a heat gradient of 27 °C–45 °C; the animals were maintained on a light–dark cycle of 7:00−19:00. All animals were well habituated to being handled for use in behavioural studies and regular health checks, though animals were rarely handled for prolonged periods. We used individuals that were experienced with being handled in order to increase the relevance of our findings to owned (pet) populations.

### 2.2. Handling and Carriage Procedure

Before each trial, the experimenter’s hands were thoroughly washed with perfume-free soap. Vivarium doors were opened slowly and smoothly, with minimal noise. If the lizard was in a hide at the start of the test, one gentle, audible tap was made on the top of the hide to alert the lizard without startling it and the hide lifted out of the way. Each lizard was removed from its vivarium by gently placing a hand on top of its body, in a loose v-grip (index and middle finger either side of head, palm down over the body), with the palm of the other hand placed underneath to support its body as the lizard was lifted out of the enclosure. The lizard was allowed to sit on the handler’s arm and/or hand while being gently secured with the other hand, placed palm down on the lizard’s back. The lizard was immediately and carefully transported into the adjacent room, containing the test arena, and either held for the requisite amount of time (Conditions 2 and 3) or placed directly into the arena (Condition 1). The lizard was placed into the arena by lowering it to the floor and allowing it to step off the hand, slowly withdrawing the hand at the same time.

Each lizard was tested once under each of the three handling conditions:

- Condition 1: Carriage Only
  Lizards in this condition were handled for the minimum amount of time possible to allow removal from their home enclosure and transport to a test arena in the adjacent room. This took no more than one minute.
- Condition 2: Five minutes of handling
  Lizards in this condition were handled for five minutes. Handling style remained consistent throughout and involved gentle restraint only as necessary to prevent escape, with free movement allowed around the experimenter’s hands and lower arms. No sudden movements, stroking or talking were undertaken, the lizards were held by the same experienced handler throughout and the handler remained still while handling the animals. They also experienced the same carriage time as mentioned in Condition 1.
- Condition 3: Fifteen minutes of handling
  Lizards in this condition were handled for fifteen minutes. Handling took place in the same way as described for Condition 2 and the lizards experienced the same carriage time as mentioned in Condition 1.

Testing took place four days per week for a period of three weeks. At least four days were allowed between testing the same lizard under different conditions. Handling condition order and order of testing within each day were counterbalanced to control for order effects (i.e., similar numbers of individuals experienced each possible order). No other studies were conducted on the animals concurrently with this one, and normal (brief) daily handling for husbandry purposes continued throughout.

### 2.3. Anxiety Testing

Novel object and novel environment tests are commonly used to assess anxiety-related behaviour in mammalian [11] and avian species [24], with typical behavioural responses including increased time spent moving (e.g., Ito et al. [25]) and reduced latency to move in less anxious individuals (e.g., Hemsworth et al. [11]). Bearded dragons have previously been shown to exhibit a behavioural response to novelty in both novel environment [5] and novel object tests [26]. Siviter et al. [26] found that lizards that had been incubated at a hotter temperature initially spent more time in close proximity to a novel object. Moszuti et al. [5] found that, although typical measures of anxiety (such as reduced locomotion) did not differ for bearded dragons when exposed to a novel vs. familiar environment, tongue flicking behaviour (incorporating both air flicks (i.e., tongue flicks directed into the air) and tongue touches (i.e., tongue flicks making direct contact with a surface) increased in response to experiencing a novel environment. This suggests that the lizards recognised and responded differently to novel environments, thus these tests represent a useful tool to assess the impact of different husbandry on behaviour and welfare.

The test arena was set up on the floor of the test room and measured 78 × 78 × 42 cm. The flooring and wall coverings were different for each trial in order to maintain novelty, with variation in both visual appearance and texture, contextual changes that we have previously found to be effective [5]. The bottom of the arena was marked with a 20 × 20 cm grid to allow for assessment of the position of the animal and with a large ‘X’ denoting the location in which each lizard was initially placed. All trials were filmed using a video camera and the recordings analysed later. The arena was maintained at 27 °C ± 1 °C throughout the experiment, which is a normal temperature for non-basking lizards. Before each trial the test enclosure was thoroughly cleaned with a Defra-approved veterinary disinfectant.

The two tests were undertaken one after the other, as follows:

- Novel environment test
  Each subject was observed from the moment it was placed in the test arena for a total of five minutes. The number of air-flicks and tongue-touches were counted and combined, as well as recording the time spent in locomotion and latency to move.
- Novel object test
  The novel object test began immediately after the novel environment test finished. The object (one of three different shapes made out of Mega Bloks^®^ (Fisher-Price, Montreal, Canada)) was placed in the test enclosure at 20 cm from the position of each lizard (approximately one lizard length). The object was chosen to be ecologically irrelevant [27] and was cleaned with disinfectant before each trial to reduce potential olfactory cues. The number of air flicks and tongue touches were counted and combined, and latency to move towards the object, latency to reach 5 cm away from the object (within reach of the lizard upon head extension) and time spent within 5 cm of the object were measured during the 5 min trial.

### 2.4. Statistical Analysis

Lizards were excluded from the study if they had entered a shed cycle; this affected three trials (three different lizards); and on two occasions, tests were stopped due to escape behaviour. This resulted in our having data for 13 lizards in the Carriage Only condition; data for nine lizards in the Five Minute Handling condition; and data for 11 lizards in the Fifteen Minute Handling condition. Response variables were the behavioural measures recorded in the novel object and novel environment tests. A General Linear Model (GLM) was used to assess each behavioural measurement, using ‘Condition’ (carriage only/5 mins/15 mins) as a fixed factor and ‘Subject’ (individual) as a random factor. ‘Order’ was initially included in each model, but was subsequently removed due to non-significance. Residual data met assumptions for parametric testing. All analyses were conducted using Minitab v17 (https://minitab.com).

## 3. Results

### 3.1. Novel Environment Test

**Figure 1:**
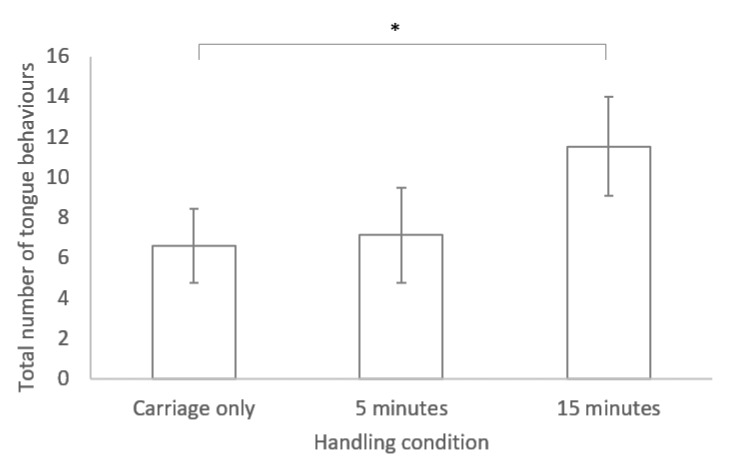
Bar chart of mean ± standard error for the number of tongue behaviours performed during the novel environment test for lizards in the three different handling conditions. * indicates a significant difference (*p* < 0.05).

No significant difference was found between any of the three handling conditions for time spent in locomotion and latency to move (see Table 1). However, a significant difference was found between handling conditions for the tongue flicking behaviour, with more tongue flicks occurring following handling for 15 min compared to the carriage only group (GLM: R2= 58.59 F2, 19 = 4.54, *p* = 0.024; see Figure 1)

### 3.2. Novel Object Test

**Figure 2:**
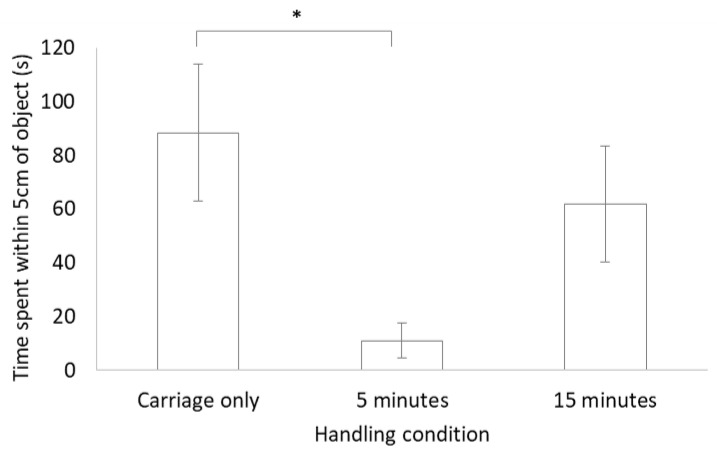
Bar chart of mean ± standard error for the time spent within 5 cm of the object during the novel object test for lizards in the three different handling conditions. * indicates a significant difference (*p* < 0.05).

No significant difference was found between each of the three handling conditions for latency to move, latency to reach within 5 cm of the object or for a combination of tongue behaviours (air flicks plus tongue touches; Table 2). There was, however, a significant difference between handling conditions for time spent within 5 cm of the object, with more time spent close to the novel object for the carriage only group compared to the group that was handled for 5 min (see Figure 2).

## 4. Discussion

Overall, we found that there appeared to be a mildly aversive effect of increased handling time on subsequent behavioural response to novelty in bearded dragons. In comparison to the Carriage Only condition when minimal handling occurred, longer durations of handling (5 min/15 min) appeared to increase anxiety-related behaviour, with handled animals showing more frequent tongue flicking behaviour when they experienced a novel environment and reduced time spent in close proximity to a novel object. This effect was observed despite the animals being very experienced with being handled (albeit briefly) and the gentle handling procedure used.

These results suggest that the handling technique applied here, although gentle and with minimal restraint compared to other studies on reptile handling (e.g., Schuett et al. [15], Moore et al. [28] and Husak et al. [29]), was mildly stressful for the lizards, resulting in some increases in anxiety-related behaviour in response to novelty. Time spent in close proximity to a novel object is a commonly used measure in a variety of species to reflect anxiety [11,30,31] and has also been previously observed in lizards that experienced different incubation temperatures [26]. The interpretation of tongue flicking behaviour as relating to anxiety in the context of exposure to a novel environment is less clear, not least because it is an order specific behaviour, occurring only in squamate reptiles [5,32], compared to more general behaviour such as changes in locomotion. Snakes, for example, can show tongue flicking in the context of exploration, particularly in response to cues from prey [32]. In a previous study that compared behavioural responses in bearded dragons to familiar and novel environments, lizards were observed to increase tongue flicking frequency in response to novelty [5]. It was postulated that the response may not be due to anxiety but a chemosensory response to novelty without an emotional driver-particularly as there were no other significant behavioural changes. However, if this were the case in the present study, no significant difference would have been expected between the ‘carriage only’ and handling conditions as the environment was novel to all individuals. We therefore suggest that, at least within this particular context, tongue flicking behaviour does reflect a stress response, and this interpretation is backed up by the complimentary behavioural change observed in the novel object test.

An apparent inconsistency of our results was that statistically significant differences were between the ‘carriage only’ and ‘15 min’ conditions in the novel environment test and between the ‘carriage only’ and ‘5 min’ conditions in the novel object test. It is not immediately clear why there was no cumulative impact of the handling experience (i.e., carriage only <5 min handling <15 min handling). Other species, for example trout, have shown increased water-borne cortisol concentrations after repeated handling compared to a single handling stress that, in turn, had higher concentrations than a no stress control [33]. One explanation could be that the two tests of novelty used in our study resulted in differential behavioural expression in response to the stress caused by handling. The assumption is that both tests would generate a similar state of anxiety (i.e., due to the presence of novelty (either object or environment)), but they could represent quite different discrete emotional contexts. It is, therefore, possible that the lack of difference between the two handling conditions (5 min and 15 min) may have arisen despite variation in the level of stress experienced. However, the important point is that the performance of the ‘carriage only’ condition was different to the ‘handling’ conditions (i.e., sustained handling versus little handling), and, in both instances, appeared to reflect reduced anxiety. Although it should be noted that, as there were no other behavioural differences between the conditions, these effects are likely to be mild.

It therefore seems that gentle short-term handling, reflecting the approach typically used by pet owners, may cause mild anxiety in bearded dragons. This is consistent with previous studies showing that handling and restraint causes stress in reptiles, including iguanas [14] and royal pythons [19]. Although Kreger and Mench [19] found no difference between gentle handling, manual restraint and container restraint in skinks, this may have been due to the gentle handling inducing considerable stress, which was then not significantly increased during more intense handling. This finding is consistent with the lack of previous habituation to handling for the animals used in that study. Other studies have found that habituation to handling reduces anxiety. For example, handling experience can have an anxiolytic effect on the subsequent behaviour of rodents in anxiety tests (e.g., Schmitt and Hiemke [34]). The animals used in our study are regularly picked up briefly and moved between rooms; however, they are less experienced with being handled for longer durations, and so it is not clear whether more experience of handing would reduce the observed anxiety or whether such handling should be avoided entirely.

In the UK, handling advice for bearded dragons is largely intended to support checks for health issues, and involves gently scooping up the lizard with both hands while supporting all four legs [35]. The RSPCA also advises not handling bearded dragons for longer than 10–15 min, although the reasoning behind this is linked to the effect on body temperature rather than the experience of stress. Our findings therefore contribute to the scientific advice underpinning guidance for handling reptiles.

## 5. Conclusions

The results of this study suggest that handling bearded dragons, even in a gentle way, may increase anxiety. However, it remains unclear how much experience animals might need in order to habituate to this. It is essential that this topic is explored in a wider range of commonly kept reptiles to help educate pet owners and encourage them to handle their animals appropriately. This will involve spending a considerable amount of time habituating pet reptiles to their owner’s presence before beginning handling, and then gradually increasing handling duration-ideally allowing voluntary approach by the reptile. Eventually, handling time might be able to be increased as the reptile becomes more comfortable with the situation, provided that the reptile in question has been habituated sufficiently. Pick-up and handling should always follow best practice, including supporting the reptile appropriately to ensure it feels safe. It should also be emphasized that different reptile species may vary in their response to handling, and individual variation may mean that while some animals can be handled, others of the same species may not.

## Figures and Tables

**Table 1:** Results of general linear model analysis to test the effect of handling condition as a fixed factor on behaviour in the novel environment test.

Behaviour	GLM Output
Tongue flicking	F_2,19_ = 4.54 (*p* = 0.024)
Time in locomotion (s)	F_2,19_ = 0.72 (*p* = 0.499)
Latency to move (s)	F_2,19_ = 0.91 (*p* = 0.418)

**Table 2:** Results of general linear model analysis to test the effect of handling condition as a fixed factor on behaviour in the novel object test.

Behaviour	GLM Output
Tongue flicking	F_2,18_ = 0.36 (*p* = 0.701)
Latency to move (s)	F_2,18_ = 0.39 (*p* = 0.682)
Latency to reach 5 cm	F_2,18_ = 0.20 (*p* = 0.820)
Time within 5 cm	F_2,15_ = 4.00 (*p* = 0.041)
